# AOJNMB changed its publication date; New Year, New Issue!

**Published:** 2015

**Authors:** S.Rasoul Zakavi

**Affiliations:** Editor-in-chief

As the New Year approaches, it is the time to review the works, efforts and life in the last year and appreciate those who helped us in our success. It is my great privilege to thank our distinguished editors, reviewers, authors as well as readers of AOJNMB who contributed enormously to the success of journal in year 2014. I hope they will continue their support of AOJNMB in the New Year 2015 too.

We published the first issue of AOJNMB in May 2013 and continued on time publication of journal every 6 months (i.e.: Oct.2013, May2014 and Oct 2014). However journals are encouraged to be published at the beginning of each period according to the calendar year and it means that for our journal with two issues per year, it is preferable to be published on the 1st of January and 1st of July each year. This policy in main citation centers lead us to change our publication date, so that we published the first issue of the 2015 on the 1st of January, and the 2nd issue will be published on 1st of July 2015.

In the year 2014, we published two issues and a supplement which included abstracts of presentations in 13th Annual General Meeting of Asian Regional Cooperative Council for Nuclear Medicine (ARCCNM) which was held on 5 and 6 of November 2014 in Osaka, Japan. The supplement issue was printed in Japan by generous support of Prof.Hatazawa and distributed among all participants of the meeting.

Most of the articles (72.3%) published in AOJNMB in previous issues were clinical studies however articles with the subjects of physics and radiopharmacy each consisted of about 15% of published articles. We received articles from 14 different countries and published 41 of them in the previous issues and our rejection rate was 31.9%. We are committed to improve quality of the journal by publishing high quality articles and we expect AOJNMB to have more impact in nuclear medicine knowledge in the future. The diversity of countries contributed to AOJNMB proves that the journal is well known in many countries. Japan, Iran and Korea outnumbered other countries for publication in AOJNMB. Also Thailand and Vietnam were more active in submission of article to AOJNMB compared to other countries in the region. Otherwise there was limited number of article submitted from Australia, India and China. This is surprising by the way and warrants further introduction and spread of AOJNMB in these countries. Although the number of published articles from different countries may partly reflect the number of research projects in nuclear medicine centers in those countries, anyhow review of nuclear medicine publications in Scopus and Pubmed had more interesting results. A search strategy developed to look for English publications in Scopus from nuclear medicine centers in year 2014 in each country. The search codes were: Affilcountry (country name) AND language (English) AND PUBYEAR = 2014 AND AFFILORG (“nuclear medicine”). Searching Scopus database with that strategy showed that 478 articles published in year 2014 from China, 338 from Korea, 306 from India, 224 from Japan, 164 from Australia, 113 from Iran and 36 from Pakistan (1-2). A similar result was obtained from searching of PUBMED (Table 1). The results are concordant with previous studies in this field (3).This information showed us that some countries like China have a very good potential for more publication in AOJNMB and re-emphasized the necessity of recognition and spread of AOJNMB in China, India and Australia.

**Table 1:** Number of published articles from Nuclear medicine departments in year 2014 in scopus and pubmed

Country	Scopus	Pubmed
China	478	591
Korea	338	331
India	306	332
Japan	224	201
Australia	164	145
Iran	113	73
Pakistan	36	20
Saudi Arabia	21	22
Thailand	12	12
Malaysia	11	7
Indonesia	3	5

AOJNMB is entering its third year of publication in 2015 and we are happy that this journal works as a platform for a completely free and open access publication of scientific results in nuclear medicine and related subjects. We believe that this free open access journal will help the scientists and physicians working in nuclear medicine by easy sharing of their knowledge and research with the rest of the world.

Finally, I would like to use this opportunity to wish a very happy and prosperous new year 2015 for all of our readers and invite them to submit their important and new research works for publication in AOJNMB.
